# Ethnogynaecological Assessment of Medicinal Plants in Pashtun's Tribal Society

**DOI:** 10.1155/2015/196475

**Published:** 2015-02-10

**Authors:** Muhammad Adnan, Akash Tariq, Sakina Mussarat, Shaheen Begum, Naser M. AbdEIsalam, Riaz Ullah

**Affiliations:** ^1^Department of Botany, Kohat University of Science and Technology, Kohat 26000, Pakistan; ^2^Department of Environmental Sciences, Fatima Jinnah Women University, The Mall Rawalpindi, Punjab 46000, Pakistan; ^3^Riyadh Community College, King Saud University, Riyadh 11437, Saudi Arabia; ^4^Department of Chemistry, Government College Ara Khel, Frontier Region Kohat 26000, Pakistan

## Abstract

The present study was designed to document detailed ethnogynaecological knowledge of selected remote regions of Pashtun's tribe in northwest Pakistan. Semistructured questionnaires were designed to collect ethnogynaecological and ethnographic data. Total of 51 medicinal plants belonging to 36 families were documented that were used by the women of studied regions for the treatment of 9 types of gynaecological complaints. Majority of the plants (19) were found used against menses followed by 11 plants each for gonorrhea and pregnancy. Bannu region has high number of gynaecological plants (22) followed by Karak (15). Women of the regions mostly used whole plants (33%) and leaves (31%) for various ethnomedicinal preparation of gynae. Fic results showed that all ailments in different areas scored high consensus ranges between 0.6 and 1.00. Majority of the female respondents (44%) were aged between 61 and 70 years, of which most were illiterate. Women in the remote regions of Pakistan have tremendous traditional knowledge in utilizing medicinal plants for their reproductive health. Plants with high Fic values should be cross-checked for their *in vitro* and *in vivo* validation. Young girls should be educated on the importance of ethnogynaecological practices to conserve this valuable knowledge.

## 1. Introduction

Medicinal plants are always an essential part of human health care system because there are major concerns about synthetic drugs' expensiveness, side effects, and toxicity. WHO reported that almost three-fourths of the world population rely on traditional medicines [1]. In the present time, it is very much essential to find some alternative medicines for the treatment of variety of ailments [2]. More than 50% of all modern clinical or allopathic drugs are of natural product origin; hence traditional medicines can play a vital role in the pharmaceutical sciences [3]. Pakistan is bestowed with a great diversity of medicinal plants. Out of 6000 flowering plant species in Pakistan, 600 species were reported for their medicinal uses [4].

Gynaecology is the medical treatment of female reproductive system (uterus, vagina, and ovaries) health. Ethnogynaecology is an emerging new branch that basically deals with the healing of ailments among tribal women, for example, abortion, menstrual trouble, leucorrhoea, antifertility, and delivery problems [5]. Sexual and reproductive health problems account for 18% of the total global burden of disease and 32% of the burden among women of reproductive age [6]. Women in the remote areas of Pakistan depend on the plants for curing various diseases including abortion, antifertility, leucorrhoea, and other menstrual troubles. They do not go to doctor; rather they depend on herbal treatment as per the suggestions of old women or traditional healers [7]. Rural women of Pakistan are frequently experiencing gynaecological problems and are more susceptible due to poor standard of living, famine, and hard physical work, even during their pregnancy. Women, locally known as “*Daiya,*” have tremendous traditional knowledge for the treatment of these diseases utilizing medicinal plants [8]. However, this knowledge is decreasing rapidly as younger generation is taking least interest in learning these valuable practices and healing techniques.

In Pakistan, there are very few studies conducted purely on ethnogynaecology. Literature is very scarce regarding traditional medicines used by rural women for the treatment of gynaecological disorders. The present study was therefore designed to document traditional plants and their gynaecological uses in the six major areas of Khyber Pakhtunkhwa Province, Pakistan. These regions are dominated by Pashtun's tribe and remote with poor infrastructure and lack of modern facilities. People of the regions have low income status and are suffering from high level of poverty [9–12]. These factors force the locals to use traditional medicine and keep the indigenous knowledge intact. The present research was therefore designed with the aim to document ethnogynaecological knowledge of plant resources and to select candidate plants for further* in vitro* investigations. The present research would be a great contribution at both national and international level for the use of traditional plants against gynaecological problems. The present research will provide baseline information for future research studies regarding phytochemistry, pharmacology, and conservation of gynaecological plants.

## 2. Materials and Methods

### 2.1. Study Area

**Figure 1:**
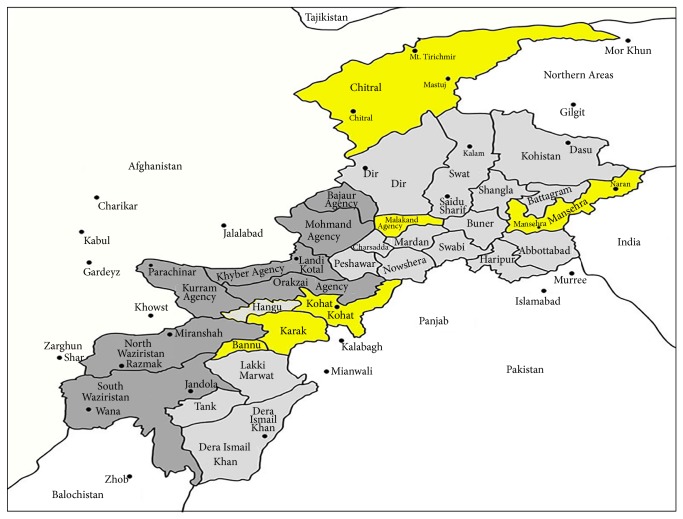
Map of the study area.

The present study was conducted in six remote areas (Bannu, Kohat, Karak, Malakand, Mansehra, and Chitral) of Khyber Pakhtunkhwa (KPK) Province, Pakistan (Figure 1). Bannu region is located in the south of KPK province and consists of 877 Km^2^ area with a population of 19,593 [9]. It lies between 32°-43 and 33°-06N latitude and 73°-20 and 70°-07E longitude. Karak is situated in the south of province with a total area of 600 km^2^ and lies between 70-40° and 71-30°N latitude and 32-48° and 33-23°E longitude [10]. Kohat is located at 33°35′13N, 71°26′29E, with an altitude of 489 m asl [13]. Malakand is located in the north of KPK between 35°10 and 35°16N latitude and 71°50 and 71°83E longitude [11]. Chitral is the largest district of KPK province with 14850 km^2^ area and lies between 35°15′06′′ and 36°55′32′′N latitude and 71°11′32′′ and 73°51′34′′E longitude with a population of about 3,20,000 [14]. Mansehra is located at 34°20′N 73°12′E of KPK a province. Majority of the population in the study regions are dominated by the Pashtun's ethnic group. All the regions are rural in nature and women of the regions are greatly dependent upon medicinal plants and forest resources for their primary health care need and for improving their livelihood.

### 2.2. Sampling and Data Collection

Data of present study was documented from January 2014 to June 2014. Prior to data collection, a brief group discussion was held with the representatives (Sherin Zaman, Faiz Ullah Khan, and Nazir Khan) of communities locally known as “*Malik*” in order to gain their consent, to explain objectives of the research study, and to assure them protection of their traditional knowledge. The selection of informants was mainly based on their rich indigenous knowledge and long term experience of utilization of plants. Total of 300 female respondents were selected in six regions with 50 informants in each area. Less number of informant selections in each area is due to the reason of cultural and religious restrictions of females. The selected respondents were local inhabitants of the regions aged between 40 and 80 years. Data was collected in the local language of the respondents and then converted into English. Semistructured questionnaires were designed to collect ethnomedicinal and ethnographic data. The informants were asked about the number of gynaecological plants known to them, their gynaecological applications, and their parts used. Ethnographic data about the age, occupation, and education of the informants were also collected. All the respondents and focal persons of the study area provided permission to publish and protect the data on traditional medicines provided by them.

### 2.3. Specimen Collection and Identification

Plants documented by key respondents were collected from home gardens and natural vegetation during field survey. The collected voucher specimens were taken to the Herbarium of Kohat University of Science and Technology (KUST), Kohat, Pakistan.

Specimen identification and confirmation were undertaken by using Flora of Pakistan and taxonomic experts. Specimens with their label were stored at the Herbarium of KUST.

### 2.4. Data Organization

The collected data on ethnogynaecological plants and ethnography of the respondents was organized using Microsoft Excel 2007 and summarized using graphical statistical methods such as percentages. The habit of the plants was categorized into 3 classes (herbs, shrubs, and trees). Reproduction of medicinal plants was classified into annual, biennial, and perennial. Plant parts were classified into leaves, roots, stem, whole plant, seeds, fruit, and flower. Gynaecological disorders were divided into 9 categories, that is, menses, gonorrhea, leucorrhoea, abortion, pregnancy, gynae, abortifacient, female impotency, and mastitis. Ages of the respondents were categorized into four groups (40–50, 51–60, 61–70, and 71–80). Education of the female respondents was classified into 5 classes, that is, illiterate, primary, middle, secondary, and university level of education. Occupation of the females was divided into only two classes (housewives and teachers).

### 2.5. Data Analysis

#### 2.5.1. Informant Consensus Factor (Fic)

Fic was used to for the general uses of plants in different study areas and to indicate plants of particular interests. Informants' consensus is the most preferred method to highlight widely used plants for a particular ailment and thus aids in the selection of plants for pharmacological and phytochemical studies [15]. Prior to using this method, illnesses were classified into categories, as high Fic plants are likely to be more pharmacologically active in comparison with low Fic value plants [16]. Fic values lie between “0.00 and 1.00.” Fic values are always greater when single plant or few plants are used by large number of informants to cure a specific disorder, while low Fic values give an indication that informants do not agree over which plant to use [17, 18]. The Fic can be calculated using the formula as follows:

(1) $$\begin{array}{c} \text{Fic}=\frac{\;\text{nur}-\text{nt}}{\text{nur}-1}, \end{array}$$

where Fic = informants consensus factor, nur = number of use citation in each category, and nt = number of species used.

## 3. Results

**Figure 2:**
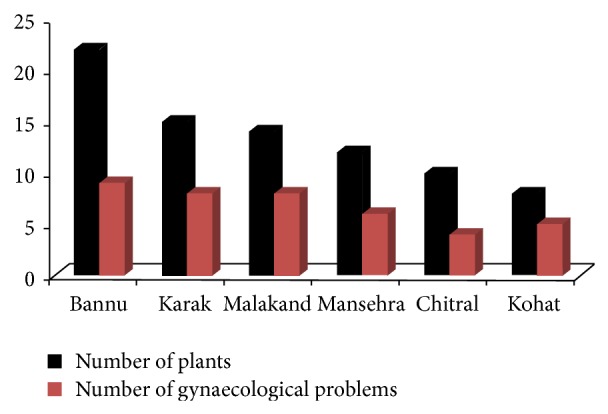
Number of plants and gynaecological problems treated in different regions of Pakistan.

**Figure 3:**
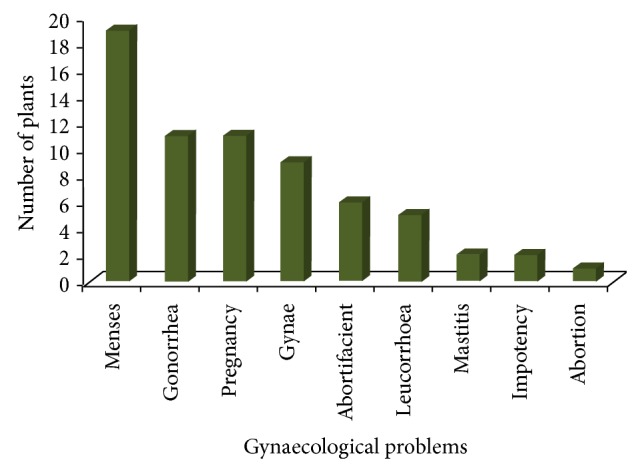
Number of plants used to treat gynaecological problems.

The present study revealed that women of studied regions used about 51 plants belonging to 36 families (Table 1). Bannu region was found with high number of gynaecological plants (22) followed by Karak (15), Malakand (14), Mansehra (11), Chitral (10), and Kohat (8). Nine types of diseases were treated in Bannu followed by eight in Karak (Figure 2). Women of the regions mostly used herbs (59%) for the preparation of ethnomedicines followed by trees (26.6%) (Table 2). It was found that majority of the plants (78%) were perennial in their mode of reproduction. Women of the regions used different plant parts for the recipe preparation but whole plant and leaves (33% and 31%, resp.) were found to be the most frequent parts used against gynaecological complaints (Table 2). Nine types of gynaecological ailments were treated in study areas. Majority of the plants (19) were found to be used against menses followed by 11 plants each for gonorrhea and pregnancy related problems (Figure 3). Fic results showed that all plants in different areas scored high consensus ranges between 0.6 and 1.00 (Table 3). Majority of the female respondents (44%) were aged between 61 and 70 years. Total of 40% informants were illiterate followed by 38% who had just primary level of education. Majority of the females (86%) interviewed were housewives followed by 14% school teachers (Table 4).

## 4. Discussion

Present study results showed that women of studied remote areas of Pakistan have strong traditional knowledge in the utilization of medicinal plants for variety of gynaecological disorders. Traditionally the rural women prefer plant medicines rather than modern medicine for their personal ailments due to lack of modern facilities in the regions. Among all studied regions, Bannu was ranked first having large number of gynaecological plants. High number of medicinal plants in the region might be associated with the prevalence of large number of gynaecological problems in the Bannu region. Nine types of ailments were found treated using ethnomedicines in Bannu region. It is a war affected region of Pakistan where traditional medicines use is a common practice [9]. Karak and Malakand regions also contain considerable number of gynaecological plants due to the greater plant diversity in the regions, rural nature, and dependency of women for their primary health care needs [11, 19].

The women of the studied regions mostly use herbs (59%) for the preparation of ethnomedicines followed by trees (26.9%). In most remote areas, medicinal herbs are the main ingredients of local medicines and considered the main lifeline and frequently first choice. The highest use of herbs gives an indication of the presence of great abundance of herb species as noticed during field visits that areas very close to houses were well covered with herbs and centuries old traditional knowledge of the healers. Common use of herbaceous plants has also been reported from other regions of Pakistan [2, 20] and parts of the world [21, 22]. Herbs can grow in variety of places like roadsides, home gardens, farmland, wild habitats and found more common in comparison to other growth forms. The highest tree species utilization might be associated with their potential to survive even during long dry seasons; thus their abundance and availability throughout the year is higher in arid and semiarid areas. The findings are in line with some studies [10, 23] while being contradictory with studies conducted elsewhere where shrubs were more frequently used [24, 25]. Variation in medicinal plants growth form might be associated with different sociocultural beliefs, ecological status, and variation in practices of traditional healers of different regions or countries. Women mostly use perennial plants (78%) for the treatment of gynaecological problems. The reason behind using perennial plants might be due to the fact that high number of herbs and trees in the studied regions are perennial in their reproduction status.

Women of studied regions use reported medicinal plants for the treatment of nine types of gynaecological ailments. Menses was found to be the most treated ailment in the studied regions. Total of 19 plants were used to treat menses related problems followed by 11 plants each for gonorrhea and pregnancy, 10 for gynae, 6 for abortifacient, 5 for leucorrhea, and 2 plants each for mastitis and impotency and single plant is used for abortion. Higher plant utilization for menses might be due to natural phenomenon associated with variety of complications such as abdominal or pelvic cramping, lower back pain, bloating and sore breasts, food cravings, mood swings and irritability, headache, and fatigue [26, 27]. Different plants have been found effective in relieving menses complications like* Justicia adhatoda*,* Schinus molle*,* Convolvulus arvensis*,* Cyperus rotundus,* and* Hypericum perforatum*. Rural women use different parts of plant to prepare ethnomedicines; however, use of specific plant part depends upon plant habit and user requirements. Traditional healers mostly prefer leaves and whole plant for the formulation of gynaecological recipes. The selection of specific plant parts suggests that these parts have strong healing potential against gynaecological disorders but these parts need phytochemical screening and pharmacological investigation in order to cross-check traditional knowledge. Present findings are in line with other studies showing leaves and whole plants as the most frequently used plant parts for the preparation of different ethnomedicines [21, 22, 28]. Whole plant harvesting is considered a destructive type of harvesting and causes population reduction of plant species.

Most of the plants were found used in more than one region for the same particular ailment; for example,* Convolvulus arvensis* was used against menses complication in Bannu, Kohat, Karak, and Malakand.* Melia azadirachta* was found to be effective against gonorrhea in Bannu and Karak.* Solanum surattense* was being used against gonorrhea and pregnancy in Malakand, Bannu, and Kohat. Such types of resemblance using similar plants for the same type of ailments in different cultures or regions provide a strong signal of bioactivity potential of the documented plant species. Informant consensus results showed high consensus values ranges between 0.6 and 1.00 for 9 disease categories in different areas. High Fic values in these regions indicate high prevalence of given gynaecological problems in these regions. According to Heinrich et al. [17], high Fic values are very useful in the selection of specific plants for further search of bioactive compounds. Most of the diseases were found to be treated with only one or two plants in the studied regions and their Fic score was also high 1.00. Such plants should further be analyzed for their phytochemical and pharmacological investigation.

Ethnographic data showed that majority of the females (44%) interviewed were aged between 61 and 70 years. These results clearly indicate that traditional knowledge is restricted to aged people in these regions due to least interest of younger generation. Total of 40% informants were illiterate in these regions followed by 38% who had only primary level of education. Only 4% respondents had university level of education, which reflects the unavailability of standard educational institutions in these areas. Literate people had less knowledge about medicinal plants as compared to illiterate people of the regions due to modernization and changing life styles. Total of 86% women were housewives while only 14% of females were school teachers that might be due to the fact that in remote areas of Pakistan women are mostly confined to homes due to variety of customs and religious restrictions. In rural areas girls are supposed to be future wives, mothers, and housekeepers; little attention is given to their formal education. Rural women have little access to education, particularly at higher levels. Even at the primary and secondary levels, access is restricted, retention rates are low, and facilities, particularly in rural areas, remain abysmal. Women are poorly represented in higher and technical education and thus have poor employment prospects. The women do not get sufficient medical treatment due to unavailability of the medical facilities. Low literacy rate, lack of medical facilities, and low income of the people are the main factors for their greater dependency on medicinal plants.

## 5. Conclusions

The present study concluded that women in remote areas of Pakistan have great dependency on medicinal plants for the treatment of different gynaecological problems. Studied regions contain considerable number of medicinal plants used by the traditional healers in different herbal formulations. Menses and gonorrhea were found to be the most prevalent complications in the studied region. Plants scoring high Fic value should be further evaluated for their phytochemical and pharmacological investigation. Ethnogynaecological knowledge is only restricted to aged women while young generation is totally ignorant. Young girls should be educated regarding the importance of traditional knowledge. Moreover, detailed studies on ethnogynaecological plants should be carried out before the extinction of this valuable knowledge.

## Figures and Tables

**Table 1:** Ethnomedicinal plants used to treat gynecological problems.

Plant families	Scientific names/voucher no.	Local names	Habit	Reproduction	Part used	Region	Gynaecological problems
Acanthaceae	*Justicia adhatoda* L. KUH-810	Baikar	Shrub	Perennial	Root, leaves	Malakand, Karak, Bannu	Gynae, abortifacient

Amaranthaceae	*Achyranthes aspera* L. KUH-811	Kurshaka	Herb	Perennial	Whole plant	Kohat, Malakand	Gonorrhea

Anacardiaceae	*Schinus molle* L. KUH-812	Toor maruch	Tree	Perennial	Bark, leaf, fruits	Bannu	Menses

Amaryllidaceae	*Allium sativum* L. KUH-813	Thoma	Herb	Perennial	Seeds	Karak	Menses
	*Allium cepa* L. KUH-814	Thrashto	Herb	Perennial	Bulb	Chitral	Gynae, menses

Brassicaceae	*Brassica campestris* L. KUH-815	Sarson	Herb	Annual	Leaves	Malakand, Kohat, Karak	Mastitis
	*Sisymbrium irio* L. KUH-816	Khelikheli	Herb	Annual	Seeds	Chitral, Karak, Bannu	Pregnancy

Cannabaceae	*Cannabis sativa* L. KUH-817	Banga	Herb	Annual	Leaves and bark	Bannu, Malakand, Mansehra	Gonorrhea, pregnancy

Celastraceae	*Gymnosporia royleana* Wall. ex M.A. Lawson KUH-818	Pataki	Shrub	Perennial	Seed	Mansehra, Karak	Pregnancy

Convolvulaceae	*Convolvulus arvensis* L. KUH-819	Pryvatay	Herb	Perennial	Whole plant	Karak, Bannu, Malakand, Kohat	Menses

Cucurbitaceae	*Citrullus colocynthis* (L.) Schrad. KUH-820	Maraginye/truh	Herb	Perennial	Roots and fruits	Bannu	Abortifacient, mastitis

Cyperaceae	*Cyperus rotundus* L. KUH-821	Delloca	Herb	Perennial	Whole plant	Bannu	Menses

Equisetaceae	*Equisetum ramosissimum* Desf. KUH-822	Jorter, horse tail	Herb	Perennial	Whole plant	Mansehra	Gonorrhea

Fabaceae	*Acacia farnesiana* (L.) Willd. KUH-823	Vilayati kikar	Tree	Perennial	Gum	Kohat	Leucorrhoea
	*Acacia modesta* Wall. KUH-824	Palosa	Tree	Perennial	Whole plant	Kohat, Karak, Bannu, Mansehra	Gonorrhea, gynae
	*Acacia nilotica* (L.) Willd. ex Delile KUH-825	Kikar	Tree	Perennial	Leaves, bark, pod	Bannu, Karak, Mansehra	Gynae, gonorrhea, leucorrhea, female impotency
	*Medicago sativa* L. KUH-826	Malkindye	Herb	Perennial	Leaves, stem	Bannu	Menses
	*Lotus corniculatus* L. KUH-827	Rub	Herb	Perennial	Whole plant	Chitral	Pregnancy

Hypericaceae	*Hypericum perforatum* L. KUH-828	Sheen chai	Herb	Perennial	Fruit, shoot	Malakand	Menses

Juglandaceae	*Juglans regia* L. KUH-829	Ghuz	Tree	Perennial	Bark	Kohat, Mansehra	Gynae

Juncaceae	*Juncus thomsonii* Buchenau KUH-830	Gawag	Herb	Perennial	Whole plant	Chitral	Pregnancy

Lamiaceae	*Mentha viridis* (L.) L. KUH-831	Podina	Herb	Perennial	Leaves	Malakand	Menses
	*Thymus serpyllum* L. KUH-832	Mervezei	Herb	Perennial	Whole plant	Bannu	Menses, gynae

Malvaceae	*Abelmoschus esculentus* (L.) Moench KUH-833	Bhindi	Herb	Annual	Fruits	Karak	Gonorrhea
	*Abutilon indicum* (L.) Sweet KUH-834	Koso beta	Shrub	Annual	Whole plant	Bannu	Leucorrhoea, gynae, gonorrhea, abortion
	*Malva parviflora* L. KUH-835	Tikalai	Herb	Annual	Leaves	Bannu	Menses

Meliaceae	*Melia azadirachta* L. KUH-836	Bakana	Tree	Perennial	Bark, fruits gum	Bannu, Karak	Gonorrhea

Myrtaceae	*Eucalyptus globulus* Labill. KUH-837	Lachi	Tree	Perennial	Leaves, oil, stem	Malakand	Menses

Nyctaginaceae	*Boerhavia coccinea* Mill. KUH-838	Insut/punara	Herb	Perennial	Whole plant	Malakand	Menses

Oleaceae	*Olea ferruginea* Royle KUH-839	Khuna	Tree	Perennial	Fruits, leaves	Malakand	Menses
	*Fraxinus xanthoxyloides* (G. Don) A. DC. KUH-840	Toor	Herb	Perennial	Bark, stem, leaves	Chitral	Gynae

Papaveraceae	*Papaver somniferum* L. KUH-841	Posat	Herb	Annual	Flower, fruit	Mansehra	Abortifacient, pregnancy

Plumbaginaceae	*Plumbago zeylanica* L. KUH-842	Chmchi pattar	Shrub	Perennial	Root	Mansehra	Abortifacient

Plantaginaceae	*Veronica agrestis* L. KUH-843	Khoso beta	Herb	Annual	Whole plant	Bannu	Menses, pregnancy

Poaceae	*Arundo donax* L. KUH-844		Herb	Perennial	Stem, rhizome	Bannu	Menses
	*Desmostachya bipinnata* (L.) Stapf KUH-845	Ghar chichona	Grass	Perennial	Whole plant	Karak	Menses

Polygonaceae	*Polygonum biaristatum* Aitch. & Hemsl. KUH-846	Howar	Shrub	Perennial	Whole plant	Bannu	Gonorrhea

Ranunculaceae	*Aconitum heterophyllum* Wall. ex Royle KUH-847	Patris, bhang dewana, sarba wali	Herb	Perennial	Latex, root	Mansehra	Gynae

Rhamnaceae	*Ziziphus mauritiana* Lam. KUH-848	Bera	Tree	Perennial	Leaves, bark, seeds	Bannu	Gynae

Rosaceae	*Crataegus songarica* K. Koch KUH-849	Ghonii	Tree	Perennial	Leaves, stem, bark	Chitral	Gynae

Rubiaceae	*Randia tetrasperma* Benth. & Hook. f. KUH-850	Mainphal	Shrub	Perennial	fruit	Chitral	Abortifacient

Rutaceae	*Zanthoxylum armatum* DC. KUH-851	Timbar	Tree	Perennial	Fruit, leaves	Mansehra	Abortifacient

Salicaceae	*Salix acmophylla* Boiss. KUH-852	Chekar	Herb	Perennial	Leaves, twigs	Chitral	Menses

Saxifragaceae	*Bergenia stracheyi* (Hook. f. & Thomson) Engl. KUH-853	Bisabur	Herb	Perennial	Leaves, roots latex	Chitral	Pregnancy

Solanaceae	*Hyoscyamus niger* L. KUH-854	Joli gao	Herb	Biennial	Leaves	Chitral	Pregnancy
	*Solanum surattense* Burm. f. KUH-855	Manraghonay/mahokri	Herb	Biennial	Whole plant	Malakand, Bannu, Kohat	Gonorrhea, pregnancy
	*Withania coagulans* (Stocks) Dunal KUH-856	Panir, panir doda	Herb	Annual	Fruits	Kohat, Karak, Bannu	Leucorrhoea
	*Withania somnifera* (L.) Dunal KUH-857	Kotilal, jangli paneer	Shrub	Perennial	Whole plant	Malakand, Mansehra, Karak, Bannu	Leucorrhea, female impotency, menses
	*Datura metel* L. KUH-858	Barbaka	Shrub	Perennial	Whole plant	Karak, Bannu	Gonorrhea

Tamaricaceae	*Tamarix aphylla* (L.) H. Karst. KUH-859	Sheen ghazz	Tree	Perennial	Leaves, bark	Karak, Bannu	Gynae

Verbenaceae	*Verbena officinalis* L. KUH-860	Koso beeta	Herb	Perennial	Whole plant	Bannu, Malakand	Pregnancy, menses

**Table 2:** General attributes of medicinal plants.

Attribute	Total number	Percentage (%)
Part use		
Leaves	16	31.3
Whole plant	17	33.3
Fruit	10	19.2
Bark	6	11.5
Root	5	9.6
Stem	4	7.6
Seed	4	7.6
Flower	1	1.9
Habit		
Herb	31	59
Shrub	7	13.4
Trees	14	26.9
Reproduction		
Annual	9	17.3
Biennial	2	3.8
Perennial	41	78.8

**Table 3:** Fic values of traditional medicinal plants for treating gynaecological problems in study regions.

Gynaecological problems	Bannu	Karak	Kohat	Chitral	Mansehra	Malakand
Menses	0.88	0.91	1.00	0.87	—	0.97
Gonorrhea	0.91	0.93	0.83	—	0.92	0.92
Mastitis	1.00	1.00	1.00	—	—	1.00
Pregnancy	0.94	0.81	—	0.90	0.72	0.72
Leucorrhoea	0.91	0.96	0.82	—	0.92	1.00
Abortion	1.00	—	—	—	—	—
Gynae	0.82	0.52	0.75	0.72	0.63	0.61
Female impotency	0.91	0.93	—	—	1.00	1.00

**Table 4:** Ethnographic data of study regions.

Ethnographic characters	Bannu	Karak	Malakand	Chitral	Mansehra	Kohat	Percentage
Age groups							
40–50	6	5	7	4	5	4	10
51–60	15	12	13	12	10	10	24
61–70	20	22	21	23	20	26	44
71–80	9	11	9	11	15	10	22
Education							
Illiterate	22	19	23	17	19	20	40
Primary	18	22	20	14	19	19	38
Middle	5	6	2	9	6	6	11
Secondary	3	2	3	6	4	4	7
University	2	1	2	4	2	1	4
Occupation							
Housewives	43	39	46	47	41	43	86
Teachers	7	11	4	3	9	7	14
